# Science as an Adventure - Lessons for the Young Scientist*

**DOI:** 10.5041/RMMJ.10001

**Published:** 2010-07-02

**Authors:** Avram Hershko

**Affiliations:** Nobel Prize Laureate in Chemistry, 2004. From the Unit of Biochemistry, The Bruce Rappaport Faculty of Medicine, and the Rappaport Institute for Research in the Medical Sciences, Technion-Israel Institute of Technology, Haifa 31096, Israel

I graduated as MD in 1965, after gaining substantial exposure to basic science in the course of my medical studies. I obtained my PhD degree in 1969, and thus, in essence, I have been actively engaged in conducting research for over half a century. It goes without saying, that to be successful in any field one has to love what one does. This is most important in experimental science, since experiments do not always work and at times the results disprove what you had been hoping for. Moments when everything comes together and you can shout “Eureka” are few and far between, and the outcomes attained might not exactly fit the starting hypothesis. However, such *unexpected results can turn out to be of greatest importance*.

In one of my first experiments during my postdoctorate period, I observed quite by accident that the enzyme involved in the degradation of the protein tyrosine aminotransferase required energy. I wondered why would the degradation of an *intracellular* protein require energy, whereas to our knowledge protein degradation *outside* of cells – e.g. the digestion of food – does not. This “accidental” observation led me to assume the existence of some kind of novel, unknown energy-dependent mechanism that governs highly selective protein degradation within cells. I was very impressed by this finding, and all my subsequent work was influenced by this one experiment. Although it had been reached fortuitously, I considered the observation to be important. It might have been mere luck that I chose to do this type of experiment early on in my career, but luck by itself would not have steered me toward further achievements. I had to *embark on serious scientific work to pursue this unique finding* – and I have been pursuing it ever since.

Another factor to which I attribute my success are the well known adages: “Mikol melamdai hiskalti” (*From all my teachers I have learned*), and “Asseh lecha Rav” (*Get yourself a Sage as your teacher*). I was blessed with parents who put their children’s education as their highest priority. Both my parents were teachers at some point in their life and they bestowed the love of learning upon their children. I was sent to good schools and I was interested in many subjects and it was difficult for me to decide what career to pursue. Again, some might call it luck or providence that I chose Medicine, but the real reason was that my brother was a medical student and I was able to inherit his textbooks. I was fortunate to be accepted to the Hebrew University–Hadassah School of Medicine in Jerusalem.

My first mentor in science was Jacob Mager. I could not have asked for a better teacher. Professor Mager was an eminent biochemist, a walking encyclopedia of medical sciences with vast experience in laboratory work to match his knowledge, and also very rigorous in the design and performance of his experiments. Every experiment had to be done with all necessary controls, and had to be repeated several times in order to confirm that the results were true results and not an artifact. I completed my PhD in Professor Mager’s laboratory and under his tutelage. From my experience in his lab I could gain a solid foundation in biochemistry as the basis for my subsequent research activity.

For my post-doctorate studies I went to the University of California, San Francisco, to the lab of Gordon Tomkins. Gordon Tomkins gave me a totally different perspective on science. He was less concerned with experimental details and rigorous controls, but was bursting with imaginative and stimulating ideas. He kept things fresh, coming up with new insights that led us to explore and experiment in ways that we had not thought of before. Thus, I was blessed to have received my initial scientific education from mentors who imbued me with a combination of rigorous scientific methodology on the one hand, complemented with magnificent ideas and unconventional imagination on the other, a truly winning combination. An imaginative scientist who can think outside the box and can then design and execute a series of experiments with all needed controls, is bound to be successful in his endeavors.

My first sabbatical term was spent in the laboratory of Irwin (Ernie) Rose at the Fox Chase Cancer Center in Philadelphia. Ernie Rose, who also greatly influenced my scientific life, is very different from both Mager and Tomkins. Ernie is a “problem-solver”, and solves most problems by analyzing them from every possible angle. His approach to science is very analytical and since I am more an intuitive type, we complemented each other very well. Ernie is also very brilliant and very critical, which might cause some people to be a bit afraid – but I liked his honesty and criticism. One should not be afraid of criticism, especially constructive criticism, because it can make any good idea a better one, and save time and effort by weeding out the not-so-good ideas.

One sound piece of advice for researchers at the beginning of their career is to *choose a subject situated on a less-traveled road*. Choosing a research topic that is “hot” or popular can be beneficial in the sense that there are many people to consult with or work with, but, on the other hand, the field may be too crowded, and you will be competing against many bright investigators who might beat you in the race to the goal.

When I came to do my post-doctorate work in the lab of Gordon Tomkin, the main subject they were working on in the lab was protein synthesis induction by steroid hormones. There were about 25 postdoctorate fellows working on different aspects of protein *synthesis*. Thus, instead of becoming yet another post-doc working on that same issue, I asked to work on a project that dealt with the *degradation* of the same protein. It was not a popular research field at the time, but I was aware of its scientific importance. Of course, not all subjects that are unique or under-researched are worth researching, and you must therefore be as certain as you can that the subject that you have undertaken is indeed scientifically important and deserves an investment of precious time and effort.

On my return to Israel after completing my postdoctorate studies, I set up my lab in Haifa at the Technion, where I have remained ever since. I continued to study the process of protein degradation and, more specifically, why is protein degradation for some proteins an energy dependent process and what is the mechanism that controls this process. To do this research I adhered to what I knew to do best, namely *classical* biochemistry. Even though newer tools were emerging at the time, such as are employed in molecular biology and molecular genetics, I felt that the proper way to elucidate a novel system was by the use of biochemical methods. In no way do I undervalue other methods, whereby great advances were made in biology through molecular biology and molecular genetics; however, when researching an unknown system where biochemical information is still unavailable, certain “cutting edge” methods are not rightfully applicable. I felt that the time-honored and true “old-fashioned” biochemical methods were the adequate way to unravel this novel protein degradation system. So, *do not shy away from NOT using the most advanced technologies – if you consider that “good old-fashioned” methods will serve your purpose.*

Using these methods, I was able, together with Aaron Ciechanover, who was a graduate student at the time, to isolate the protein ubiquitin. At the time we did not know what the role of ubiquitin was, but we knew that it was essential for energy-dependent protein degradation. Through collaboration Aaron Ciechanover, additional graduate students of mine and Ernie Rose, we found that ubiquitin is covalently bound to proteins that are destined for destruction, a sort of protein “death tag”. It is the process of linking the ubiquitin to the protein for destruction that requires energy, which explained my original observation, namely, that cellular protein degradation is an energy-dependent process.

At about that time, I told Ernie Rose how limited and small Israeli research grants were. Ernie suggested that I should apply for a foreign research grant from the NIH to support my work in Israel. I was inclined to do a couple of more experiments rather than devoting time to write a grant application, but Aaron Ciechanover who was a natural manager already as a graduate student, pushed me into a chair and commanded: “You write the NIH grant application – NOW!”. This I did … and got the grant, the first of five consecutive grant periods supported by the NIH. It saved the situation in our Haifa laboratory at a very critical time. I am very grateful to the NIH for supporting my work and also to Aaron for forcing me to write the initial grant application*. It is important to have a loyal and dedicated team to work with!*

In the course of the next 10 years our laboratory succeeded to isolate, purify and define different components of the ubiquitin system, working mainly by biochemical techniques. We were then able to reconstruct diverse components of the ubiquitin system, which provided us with a better understanding of the mechanism of action of this system. After gaining this initial knowledge and insight into the ubiquitin system, molecular biology techniques were used to obtain the broader picture of this system.

Since 1990, I have been working on the role of the ubiquitin system on cell cycle control but other investigators have found that ubiquitin is essential also in other basic and important cell processes, such as gene expression, signal transduction and others. The ubiquitin system in plants targets numerous intracellular regulators that have central roles in hormone signaling, regulation of chromatin structure and transcription, responses to environmental challenges, and fighting pathogens.

Many diseases have been linked to alterations of the ubiquitin system in cells. Neurodegenerative diseases such as Parkinson’s and Alzheimer’s, mental retardation, hypertension, and many types of cancer have been linked to defects in the ubiquitin system. There already is a first generation drug called Velcade, which targets the proteosome which is part of the ubiquitin protein degradation system in the cell. Although the proteosome is not the most specific part of the ubiquitin system, this drug was found to be helpful in treating Multiple Myeloma and is presently under investigation for treating certain other types of cancer.

From what I have told you so far, you will recognize that if you set out with a genuine interest in your research subject, your science is well founded and your approach to basic research is thorough and rigorous, you enhance the prospects of obtaining valuable results and an outlook for their future application. It is very satisfying for me to see that my work in basic science is being used to fight disease and that many people will benefit from this research.

I am well aware that science is not all glamor and prizes. There are many tasks that have to be carried out in order to continue doing research. Grant writing is not one of my favorite activities and neither are writing papers. I understand that both are necessary to perpetuate research, but we should not forget that it is *the excitement of doing novel science and discovering new things* that got us into this field in the first place. If our excitement dims during our career, it will not only be our happiness that will diminish, but the quality of our work will be affected as well. One way I keep this excitement going is by not giving up bench-work. I always get excited when I actually do the experiments myself, testing out my hypothesis – and when I get results that are unexpected I get even more excited. Even after all these years, I find great satisfaction in doing the nitty-gritty science work by my own hands. I also derive great satisfaction and pleasure from being surrounded by bright and dedicated research teams, students and colleagues, without whom I would not have been able to accomplish what I did. I would like to take this occasion to thank these dedicated people: first and foremost Judith Hershko (my wife), Dvora Ganoth, Hanna Heller, Ester Eytan, Sarah Elias and Clara Segal. My gratitude and appreciations also go to all my graduate students (who are too numerous to be listed here) including Aaron Ciechanover, and to all my associates who assisted me in countless ways.

In a nutshell, my advice is as follows: Find a subject that is not yet in the mainstream but does appear important and is interesting to you. Seek out excellent mentors and learn from all of them. Do not dismiss data that do not conform with your current theory, since fortuitous findings may be most important. Use whatever technique in experimentation that will best fit your needs, regardless if it is considered “old-fashioned”. Conserve and preserve your excitement and enthsiasm about science, and never stop getting your hands “dirty” and doing experiments yourself – which I have found to be a great booster to fun and excitement when “doing science”.

## Figures and Tables

**Figure 1.**
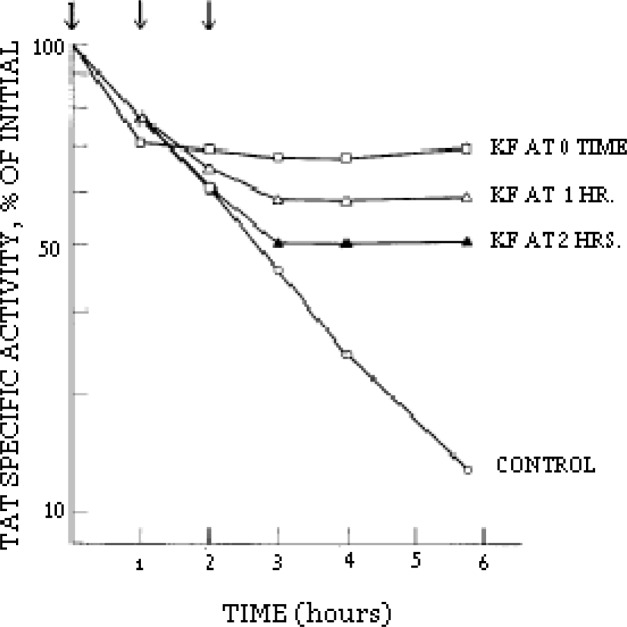
Degradation rate of tyrosine aminotransferase (TAT) is halted by inhibition of cellular energy with potassium fluoride. Arrows indicate time of K-fluoride addition to KF samples. (Hershko & Tomkins. J Biol Chem 1971;246: 710–4).

**Figure 2.**
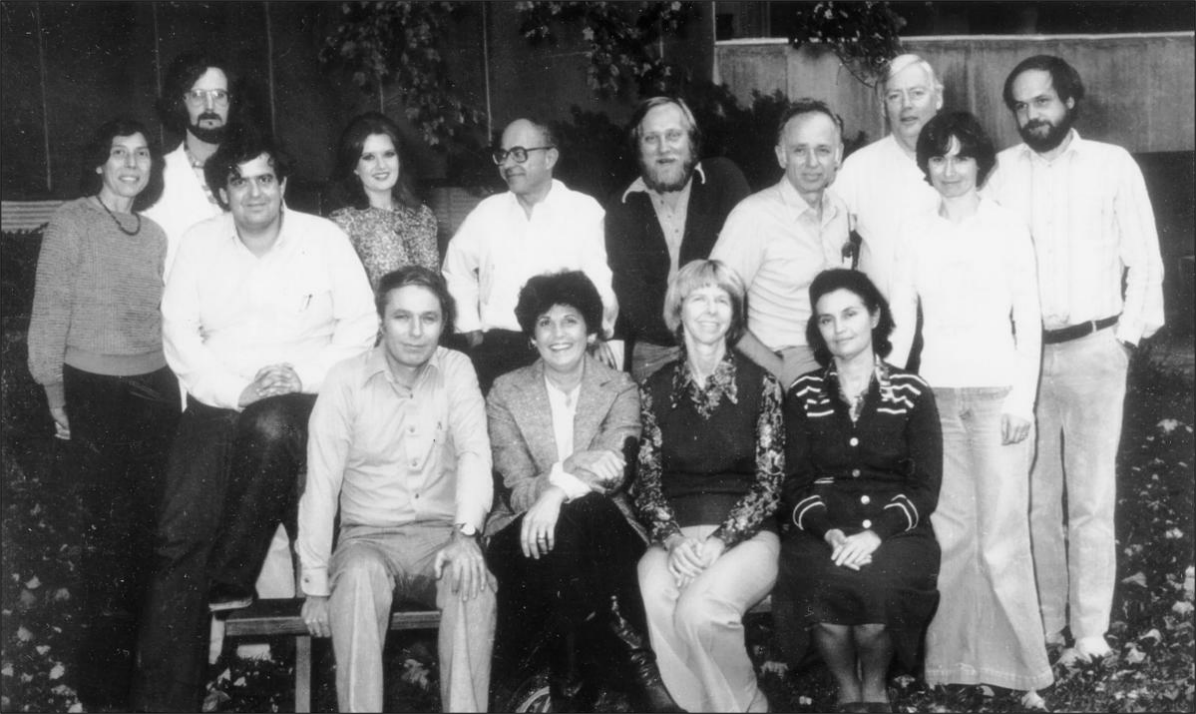
The research team at the Fox Chase Cancer Center, Philadelphia, 1979.

**Table 1.** Lessons from my life in science (thus far).

It is very important to have good mentors, you cannot learn how to do good science just from reading the literature. Find an important subject that is not yet interesting to others, otherwise the big guys will get there before you! Do not go with the mainstream. Accidental observations may be the most important ones. Grab your luck! Use whatever experimental approach is needed for your objective; it may not necessarily be the most fashionable (“state-of-the-art”) technology. Have a lot of excitement and fun in science – this is how discoveries are made! Never leave bench-work, and you shall continue to get a lot of excitement and fun.

**Table 2.** Some roles of ubiquitin-mediated protein degradation.

Control of cell division Signal transduction Regulation of gene expression Responses to inflammation and immunity Embryonic development Apoptosis

**Table 3.** Involvement of the ubiquitin system in diseases

Cancer (many types) Neurodegenerative diseases: Parkinson’s disease; Alzheimer’s disease; Huntington’s disease Mental retardation Hypertension

